# Identification and *in vitro* enzymatic activity analysis of the *AOP2* gene family associated with glucosinolate biosynthesis in Tumorous stem mustard (*Brassica juncea* var. *tumida*)

**DOI:** 10.3389/fpls.2023.1111418

**Published:** 2023-02-22

**Authors:** Bing Chen, Yu Liu, Chunfang Xiang, Dandan Zhang, Zhuoyu Liu, Yihua Liu, Jingjing Chen

**Affiliations:** School of Life Advanced Agriculture Bioengineering, Yangtze Normal University, Chongqing, China

**Keywords:** *Brassica juncea*, glucosinolate, *BjuAOP2*, expression pattern, prokaryotic expression, activity analysis

## Abstract

The major enzyme encoded by the glucosinolate biosynthetic gene *AOP2* is involved in catalyzing the conversion of glucoiberin (GIB) into sinigrin (SIN) in Brassicaceae crops. The *AOP2* proteins have previously been identified in several Brassicaceae species, but not in Tumorous stem mustard. As per this research, the five identified members of the *AOP2* family from the whole genome of *Brassica juncea* named *BjuAOP2.1*-*BjuAOP2.5* were found to be evenly distributed on five chromosomes. The subcellular localization results implied that BjuAOP2 proteins were mainly concentrated in the cytoplasm. Phylogenetic analysis of the AOP2 proteins from the sequenced *Brassicaceae* species in BRAD showed that *BjuAOP2* genes were more closely linked to *Brassica carinata* and *Brassica rapa* than *Arabidopsis*. In comparison with other *Brassicaceae* plants, the *BjuAOP2* members were conserved in terms of gene structures, protein sequences, and motifs. The light response and hormone response elements were included in the *BjuAOP2* genes’ cis-regulatory elements. The expression pattern of *BjuAOP2* genes was influenced by the different stages of development and the type of tissue being examined. The BjuAOP2 proteins were used to perform the heterologous expression experiment. The results showed that all the five BjuAOP2 proteins can catalyze the conversion of GIB to SIN with different catalytic activity. These results provide the basis for further investigation of the functional study of *BjuAOP2* in Tumorous stem mustard glucosinolate biosynthesis.

## 1 Introduction

*Brassica juncea* var. *tumida* (tumorous stem mustard, TSM) is an important vegetable crop of the Brassica genus of the Cruciferae family that originated in China. The enlarged fleshy stems of TSM are the raw material for Fuling Preserved Szechuan Pickle and can be eaten fresh. Approximately 130 glucosinolates (GSLs), an important class of secondary metabolites that are sulfur- and nitrogen-rich and found primarily in cruciferous plants, have been identified (Blažević et al., 2019; Nguyen et al., 2020). GSLs undergo hydrolysis reactions under their degradation enzyme, myrosinase, to produce products such as isothiocyanates, thiocyanates, and acetonitrile (Chen et al., 2020). The afore-mentioned products are involved in anti-cancer and anti-bacterial (Augustine and Bisht, 2015; Soundararajan and Kim, 2018) processes, resistance to herbivore feeding, pathogenic microbial infestation (Clay et al., 2009; Li et al., 2014; Chen et al., 2020), and the formation of specific flavors in cruciferous vegetables (Engel et al., 2006). The biosynthetic precursors of GSLs include amino acids such as methionine, tryptophan, phenylalanine, and leucine. These amino acids form side chains of different lengths through an extension pathway. GSLs can be categorized as aromatic, aliphatic, and indole types as per the derivation sources of the side chains (Halkier and Gershenzon, 2006). The highest content in TSM is 2-propenyl glucosinolate (sinigrin, SIN), the major aliphatic GSLs present in *B. juncea* (Li et al., 2011). TSM with high SIN content had a pronounced spicy taste (Mazumder et al., 2016). Therefore, revealing the synthesis process and molecular regulation mechanism of SIN is crucial to improve the flavor of TSM and optimize the GSL fraction in TSM.

Aliphatic GSL biosynthesis in *Arabidopsis thaliana* comprises three independent steps: elongation of the precursor amino acid-based side chain, core structure formation, and side chain secondary modification (Grubb and Abel, 2006; Sønderby et al., 2010). The current research indicates the involvement of almost 15 transcription factors and 64 structural genes in GSL biosynthesis (Li et al., 2018; Harun et al., 2020; Mitreiter and Gigolashvili, 2021). GSL-Elong is the key site in the side chain elongation step. Three genes, MAM1 (methylthioalkylmalate synthases 1), MAM2, and MAM3, located in a tandem arrangement at this locus (Kroymann et al., 2001; Kroymann et al., 2003; Textor et al., 2007). The enzyme known as MAM1 and MAM2 catalyzes the condensation reaction of the first three elongation cycles whereas MAM3 mediate the condensation reaction of all six elongation cycles (Textor et al., 2007). The functional polymorphism of the MAM gene results in differences in the structure of the GSL among different ecotypes of *Arabidopsis thaliana* (Wittstock and Halkier, 2002). The cytochrome P450 homolog *CYP79* gene family, *CYP83* gene family, and *UDP-glucosyltransferase 74B1* (UGT74B1) are key genes in the core structure formation step (Grubb et al., 2004; Mikkelsen et al., 2004). The flavin-containing monooxygenases (GSL-OX), 2-oxoglutarate-dependent dioxygenases (GSL-AOP), and 2-oxoacid-dependent dioxygenase (GSL-OH) sites during modification of the GSL side chain determine the diversity of GSL species (Kliebenstein et al., 2001a; Hansen et al., 2008). GSL-AOP includes two closely linked genes, GSL-ALK and GSL-OHP (Kliebenstein et al., 2001a). GSL-ALK (*AOP2*) and GSL-OHP (*AOP3*) genes, respectively, catalyze the transformation of methylsulfinyl GSLs into alkenyl GSLs and hydroxyalkyl GSLs (Kliebenstein et al., 2001b). In addition, the enzymes encoded by the *AOP2* gene can catalyze the conversion of beneficial GSL glucoraphanin (GRA) with anticancer activity to gluconapin (GNA) and glucoiberin (GIB) to SIN, thus affecting the taste and flavor of cruciferous vegetables.

The *AOP* gene family in *Arabidopsis thaliana* includes *AOP1*, *AOP2*, and *AOP3* (Kliebenstein et al., 2001a). The *AOP1* gene may be the ancestor of the other two genes, but its function needs to be further characterized (Neal et al., 2010). *AOP* genes greatly vary in their expression patterns in various *Arabidopsis thaliana* ecotypes and under different culture conditions. The *AOP2* is expressed in L*er* (Landsberg), but *AOP3* is absent, while Cvi (Cape Verde Islands), depicts the expression of *AOP3*, but *AOP2* is absent (Kliebenstein et al., 2001a). In addition, an ecotype in which both genes are expressed simultaneously has not been found yet. Studies in *Arabidopsis thaliana* and *Brassica napus* suggested that inactivation of the *AOP2* gene promotes the accumulation of large amounts of GRA in plants to produce higher amounts of sulforaphane with anticancer activity (Neal et al., 2010). Conversely, increased expression of the *AOP2* gene causes the GNA and SIN content to increase in plants (Neal et al., 2010; Liu et al., 2012). *In vitro* activity analysis of *Arabidopsis thaliana AOP2* protein by Kliebenstein et al. suggested that the GRA could be converted to GNA through an induced *AOP2* protein solution (Kliebenstein et al., 2001b).

Brassica, a genus of economic and nutritional importance in the Brassicaceae family, consists of six species involving three diploids: *B. rapa* (2n=20, AA), *B. oleracea* (2n=18, CC), and *B. nigra* (2n=16, BB). Mutual hybridization and continuous selective evolution among the three yielded three allotetraploids: *B. carinata* (2n=34, BBCC), *B. juncea* (2n=36, AABB), and *B. napus* (2n=38, AACC). The number and expression pattern of *AOP* genes in Brassica vary from species to species. For example, the *AOP2* gene in both *B. rapa* and *B. oleracea* has three homologs (Gao et al., 2004; Liu et al., 2014). Two and four homologs of the *AOP2* gene were found in the genomes of cabbage mustard and oil-mustard, respectively (Augustine and Bisht, 2015; Wu et al., 2017). The *BoAOP2* gene in *B. oleracea* can be expressed normally to catalyze the degradation of GRA into GNA (Zheng et al., 2022). However, there is a nonfunctional *AOP2* allele in broccoli with a two-base deletion on the exon, which in turn influences the accumulation of glucoraphanin products (Li and Quiros, 2003). Liu et al. reported that *B. oleracea* contained another non-functional *BoAOP2*. The translation termination caused by the premature termination codon functioning of this gene also caused the accumulation of glucoraphanin products (Liu et al., 2014). The presence of three alleles of *AOP2* in *B. rapa*, all with catalytic activity, was found to be tissue expression-specific (Wang et al., 2011). Augustine et al. documented a significant reduction in the amount of SIN in transgenic plants than in wild-type plants by constructing an *AOP2* silencing vector in oil mustard (Augustine and Bisht, 2015). A recently conducted study in pennycress revealed that the SIN content was significantly reduced in the wild-type F_2_ population versus the *AOP2* mutant (Chopra et al., 2020). Therefore, the *AOP2* gene is a bridge between beneficial and deleterious GSLs. Although several relevant studies have been conducted on this gene in *B. oleracea* and *B. rapa*, the mechanism that underlies its functions in TSM has not been studied in detail. Therefore, elucidating the mechanism of *AOP2* genes in GSL synthesis and degradation remains a great challenge due to the complexity of the Brassica crop genome (Malhotra and Bisht, 2020).

In this study, five genes homologous to *Arabidopsis thaliana AOP2* were cloned using the allotetraploid crop TSM (2n=36, AABB) as plant material. In addition, the protein’s physicochemical properties, gene structure, phylogenetic tree, promoter cis-acting elements, subcellular localization, and gene expression were comprehensively analyzed. The gene functions were initially verified using *in vitro* experiments. This study revealed the role of individual *BjuAOP2* genes in the process of SIN synthesis in TSM and the expression differences among different copies. Overall, the data obtained in this research acts as a foundation for revealing the molecular mechanism of SIN synthesis regulation by *BjuAOP2* genes in *Brassica juncea* and offer novel insights into improving the GLS fraction of TSM at the molecular level.

## 2 Materials and methods

### 2.1 Sample sources

The TSM high-generation selfing line B186 was sown in the Yangtze Normal University squash trial site in the fall of 2021 after germination. Per the normal growth requirements of TSM, field management was performed during growth. The leaves of B186 were collected at the 4-leaf stage for *BjuAOP2* cloning, and the expanded tumorous stems of B186 were collected at 4, 7, 10, 13, 16, 19, and 22 weeks after sowing to analyze the expression pattern of *BjuAOP2* at different developmental stages. In addition, roots, stems, leaves, tumorous stems, flowers, and siliques of B186 were collected at the shooting stagegib for analysis of the gene’s tissue expression pattern. Each sample had three biological replicates that were collected and placed in liquid nitrogen and kept at -80°C for subsequent use.

### 2.2 Identification of the *BjuAOP2* gene family

The protein sequence of the *Arabidopsis thaliana AOP2* gene was retrieved online from TAIR (http://www.arabidopsis.org/). Subsequently, a BlastP search was performed in the Brassica genome database (http://brassicadb.cn/#/BLAST/) to search for amino acid sequences and nucleic acid sequences of TSM *AOP2* gene family members. In addition, HMMsearch (Johnson et al., 2010) was used to identify the 2OG-FeII_Oxy (PF03171) and DIOX-N(PF14226) structural domains for all possible *AOP2* genes. Finally, conserved structural domain confirmation was performed through NCBI-CDD (https://www.ncbi.nlm.nih.gov/Structure/cdd/wrpsb.cgi) in NCBI to exclude sequences without typical structural domains.

### 2.3 Cloning and sequence analysis of the *BjuAOP2* gene

Total RNA of TSM leaves was isolated with Tiangen RNA Extraction Kit TRNzol Universal Reagent (Tiangen Biotech, Beijing, China) and with a subsequent reversal to single-stranded cDNA using TransScript One-Step gDNA Removal and cDNA Synthesis SuperMix kit (TransGen Biotech, Beijing, China). The designing of the primers (**Supplementary Table S1**) and amplification of the full-length CDS sequences using Primer premier 6.0 software was the next step, using the *BjuAOP2* gene sequence obtained from the stem mustard database as a template. The PCR reaction system and reaction procedure were performed according to the instructions of TransStart FastPfu DNA Polymerase from TransGen, and the PCR product recovery and purification were performed according to the instructions of the TransGen Gum Recovery Kit (EG101). In addition, the PCR-recovered products were homologously cloned with the vector pTF101-GFP using the homologous recombination method of the pEASY-Basic Seamless Cloning and Assembly Kit (TransGen Biotech, Beijing, China) from TransGen. Subsequently introducing the recombinant vector into *E. coli* receptor DH5α, plasmids were extracted from 12 positive clones for validation and sequencing. The protein sequences encoding the *BjuAOP2* gene obtained by sequencing were subjected to multiple-sequence alignment with the *AOP2* protein sequences of *Arabidopsis thaliana* using DNAStar 7.1 software. The CDS length and amino acid numbers of the *BjuAOP2* gene were obtained from the clone sequencing results. Data such as chromosome position was obtained from the reference genome. Isoelectric points and molecular weights were obtained from the pI/Mw calculation tool on the website ExPASy (http://www.expasy.org/tools/). The prediction of subcellular localization was obtained using online tool BUSCA (http://busca.biocomp.unibo.it/).

### 2.4 Phylogenetic tree construction of the *AOP* gene family in Brassicaceae crops

Genomic data of 19 sequenced cruciferous species were obtained from The Brassicaceae Genome Resource (http://www.tbgr.org.cn) (Liu et al., 2022). The protein sequence of the *Arabidopsis thaliana AOP* gene was accessed at the TAIR website as a reference sequence and the *AOP* family gene sequence was obtained using the Quick Find Best Homology tool in TBtools software (Chen et al., 2020). Clustal W was employed to carry out the multiple-sequence alignment of *AOP* family proteins from sequenced species in the Brassicaceae family in MAGE 11 software. Subsequently, utilizing the neighbor-joining (NJ) method phylogenetic trees were drawn, with the Bootstrap method set to 1000 (Tamura et al., 2021). The online tool, GSDS (http://gsds.cbi.pku.edu.cn/index.php) was employed for mapping the intron-exon structure pattern of the *AOP* gene. The analysis of the conserved motif of the gene was performed using the MEME (http://meme-suite.org/) online tool. The upstream promoter sequences (1.5-kb) of each gene of the identified *AOP* family were accessed at the Brassica genome website (http://brassicadb.org) with the promotor-bound cis-acting elements were examined by the software PlantCARE (http://bioinformatics.psb.ugent.be/webtools/plantcare/html). Schematic diagrams of all the above analysis results were illustrated utilizing the software Domain Illustrator software (http://dog.biocuckoo.org/) (Ren et al., 2009).

### 2.5 Protein subcellular localization of *BjuAOP2*

Protein subcellular localization of *BjuAOP2* was performed by transient expression in tobacco leaf epidermal cells. *Agrobacterium tumefaciens* GV3101 containing the *ProCAMV35S::GFP::BjuAOP2* vector plasmid was activated and enlarge-cultivated. Subsequently, the organisms were collected, resuspended in a resuspension solution (OD600 almost 0.5), left for 2-3 h, and introduced into the lower epidermis of 3-4 weeks old tobacco leaves with a syringe. Observations were made after 3 days of transformation. The leaves transformed with empty pTF101-GFP were used as control. In addition, laser confocal microscopy (Nikon, Tokyo, Japan) was utilized for the observation of GFP fluorescence with excitation light at 488 nm and emission light at 510 nm. Furthermore, chloroplast autofluorescence showed excitation light at 640 nm and emission light at 675 nm.

### 2.6 Prokaryotic expression and *in vitro* enzyme activity assay of the *BjuAOP2* gene

The design of the primers and amplification of full-length CDS sequences were executed through Primer premier 6.0 software, using the *BjuAOP2* gene sequence obtained from the stem mustard database as a template (**Supplementary Table S2**). The pET-32a expression vector (Novagen. Madison, WI, USA) was constructed using the full-length CDS sequence of the gene through homologous recombination with the pEASY-Basic Seamless Cloning and Assembly Kit (TransGen Biotech, Beijing, China). Subsequently, plasmids were extracted from 12 positive clones for validation and sequencing. The correctly sequenced recombinant plasmids for the prokaryotic expression of the gene were transformed into *Escherichia coli* strain Transetta (DE3). The recombinant pET vector contains the T7 promoter and capable of expressing a fusion protein containing thioredoxin. The *E. coli* DE3 strain containing prokaryotic expression of the recombinant plasmid was incubated in LB medium at 37°C while also being shaken until OD600 was approximately = 0.6. Then, a final concentration of 0.5 mM was obtained by adding IPTG, and the expression of recombinant protein was induced by shaking the bacteria overnight at 16°C. The bacteria were isolated after induction by centrifuging the *E. coli* broth for five minutes at 6000 rpm/min. These bacteria were then suspended in PBS and placed on an ultrasonic cell crusher for 3 min at 100MHz (5-second on-10-second off cycle). The obtained cell-disrupted solution was centrifuged (12000 rpm/min and 20 min) for supernatant collection. Subsequently, the supernatant was purified by fusion protein according to the instruction procedures of Ni IDA Beads 6FF Kit (Changzhou Smart-Lifesciences Biotechnology, Changzhou, China). Finally, the purified fusion protease was detected by SDS-PAGE electrophoresis.

Kliebenstein et al. (2001a) validated the *Arabidopsis thaliana AOP2* protein by *in vitro* experiments. In this procedure, the same method was used to validate the function of *BjuAOP2* protein in *Arabidopsis thaliana*, with a slight modification of the enzyme activity assay. The procedure was as follows: 400 μL of purified *BjuAOP2* protein solution, 10 mM ascorbate, 15 mM α-ketoglutarate, 200 mM sucrose, 200 μM FeSO4, and 100 μL GIB were added to five 10 mL centrifuge tubes (total reaction volume: 4 mL). A control 10 mL centrifuge tube was added with 400 μL ddH2O instead of purified *BjuAOP2* protein solution, and the other components remained unchanged. Subsequently, the six centrifuge tubes were placed on a shaker at 28°C (110 rpm) for 4 hours. The reaction solution was purified by desulfurization and subjected to high-performance liquid chromatography (HPLC) to analyze the GSL fraction

### 2.7 GSL extraction and HPLC analysis

GSLs were extracted and assayed per the prior procedure (He et al., 2002). First, 1 mL of the enzyme-activated reaction solution was transferred to a DEAE Sephadex A-25 column for overnight desulfurization at room temperature using lipase sulfate (Sigma, E.C. 3.1.6.) and eluted twice with 750 µL of deionized water. The filtration of the eluent was carried out through a 0.22 μm Hydrophilic polyethersulfone (PES) Syringe Filter (Shanghai Anpel) for HPLC analysis. The elute, the external standard 3-GIB, and SIN were analyzed by HPLC.

### 2.8 Quantitative real-time fluorescence analysis

Total RNA was obtained from different tissues and developmental stages of tuberous mustard through TRNzol Universal Total RNA Extraction Reagent from Tiangen Biotech (Beijing, China). cDNA was synthesized based on the CDS sequences obtained by homologous recombinant cloning and sequencing using Primer premier 6.0. The afore-mentioned software designed real-time fluorescence quantification primers (**Supplementary Table S3**). The reaction system was as follows: 2×SuperReal PreMix Plus 10 μL; upstream and downstream primers, 0.5 μL each; cDNA template, 2 μL; ddH2O, 7 μL. In addition, a three-step amplification method was used: 95°C pre-denaturation for 5 min; 95°C for 30 s, 58°C for 30 s, and 72°C for 30 s (40 cycles). Dissociation curve: 65-95°C (0.5°C increase per cycle) for 5 s (1 cycle). The entire fluorescent quantitative PCR reaction was performed on a Roche Light Cycler 480 instrument. The proportionate expression of the five *BjAOP2* was calculated using the 2^-ΔΔCT^ calculation method with actin as an internal reference gene (Livak and Schmittgen, 2001). Technical and biological replicates, three each were utilized in each experiment.

### 2.9 Statistical analysis

One-way analysis of variance (ANOVA), significance analysis, and Duncan’s multiple-comparison were performed on the experimental data using SPSS analysis software, with a significance threshold of p-value < 0.05.

## 3 Results

### 3.1 Identification and cloning of *BjuAOP2* gene family members

BLAST alignment of the *Arabidopsis thaliana AOP2* gene protein sequences with the TSM database was performed. Subsequently, the 2OG-FeII_Oxy (PF14226) structural domain was identified for all possible *AOP2* genes. In total, five members of the gene family distributed in different chromosomes, named *BjuAOP2.1-BjuAOP2.5*, were identified from the TSM genome database (**Table 1**). The CDS sequences of the genes mentioned above were obtained by PCR using the cDNA obtained by reverse transcription as a template. *BjuAOP2* genes have CDS sequences in the length range of 1284-1428 bp, with encoding amino acid numbers between 427-475 aa, molecular weight ranging from 46.8 to 55.2 kDa, and isoelectric point variation ranging from 4.77 to 5.66. The optimal prediction of subcellular localization is nucleus.

**Table 1:** Basic information of the *BjuAOP2* genes.

Gene ID	Rename	chromosome	Genome location		Gene length/bp	Number of amino acids/aa	Molecular weight/kDa	isoelectric point	Subcellular localization
			Start	End					
BjuVB05G45660	BjuAOP2.1	B05	46966364	46970953	1299	432	47.6	4.77	nucleus
BjuVA09G02070	BjuAOP2.2	A09	1311307	1313569	1320	439	47.9	4.89	nucleus
BjuVA02G29850	BjuAOP2.3	A02	17116725	17119697	1284	427	46.8	4.95	nucleus
BjuVB08G37450	BjuAOP2.4	B08	28215201	28217478	1428	475	52.2	5.66	nucleus
BjuVA03G30000	BjuAOP2.5	A03	14204864	14211046	1323	440	48.3	4.90	nucleus

**Figure 1:**
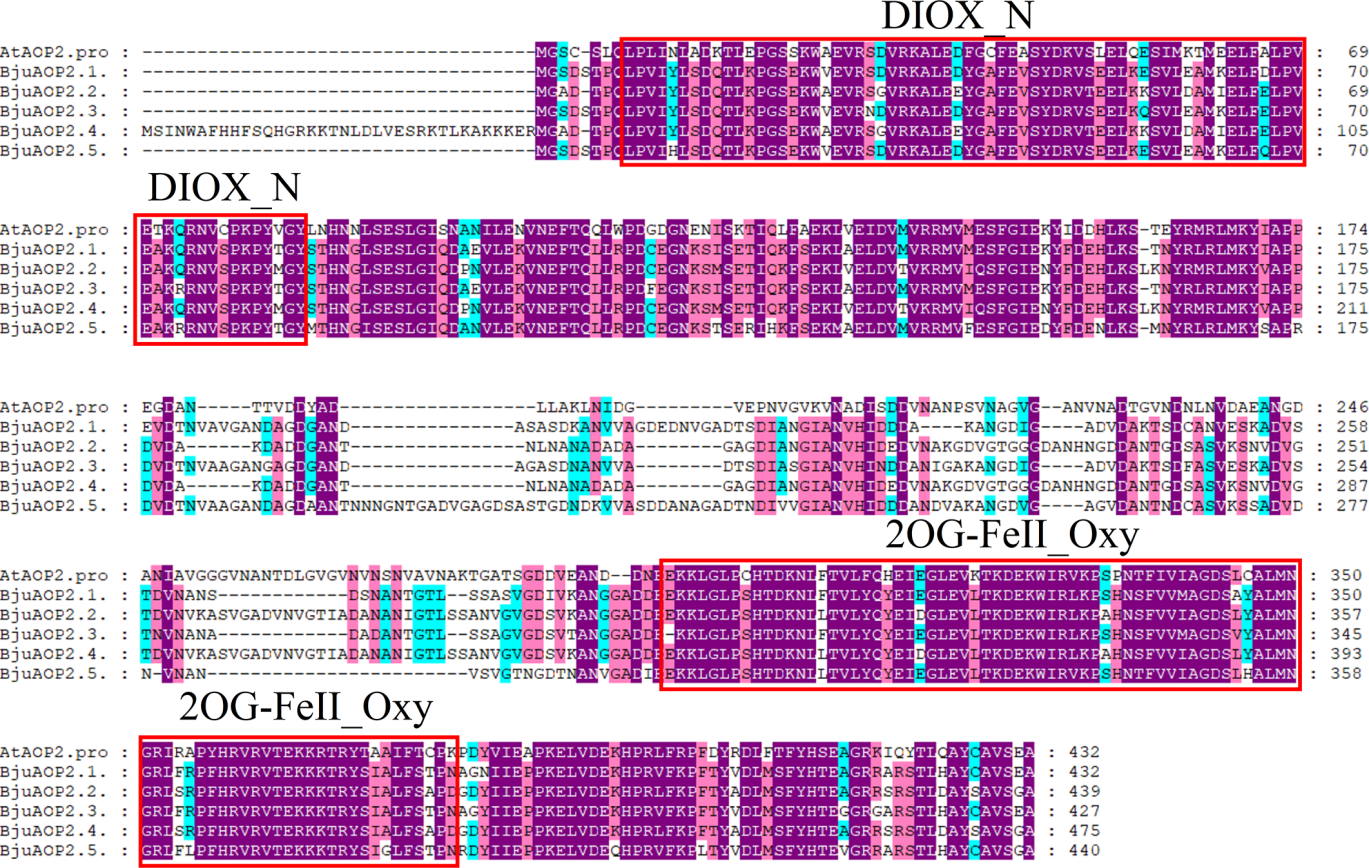
Multiple-sequence alignment of Tumorous stem mustard BjuAOP2 and AtAOP2 protein sequence. Multiple alignments were performed using the MEGA11 software. The red solid boxes represent the structural domains of 2OG-FeII_Oxy and DIOX-N. The purple, blue and pink shading indicates 100%, 80% and 60% conserved percent, respectively.

Multiple-sequence alignment with the *Arabidopsis thaliana AOP2* gene (**Figure 1**) depicted that the *BjuAOP2* gene family’s five members contain the key structural domains, DIOX-N and 2 OG-Fell-Oxy, and the sequences in this range are highly conserved. However, the protein sequence similarity of the middle part of these five members is low, leading to the divergence of gene functions among the members.

### 3.2 Phylogenetic, gene structure, motif and cis-acting regulatory elements analysis of the *BjuAOP2* gene family

**Figure 2:**
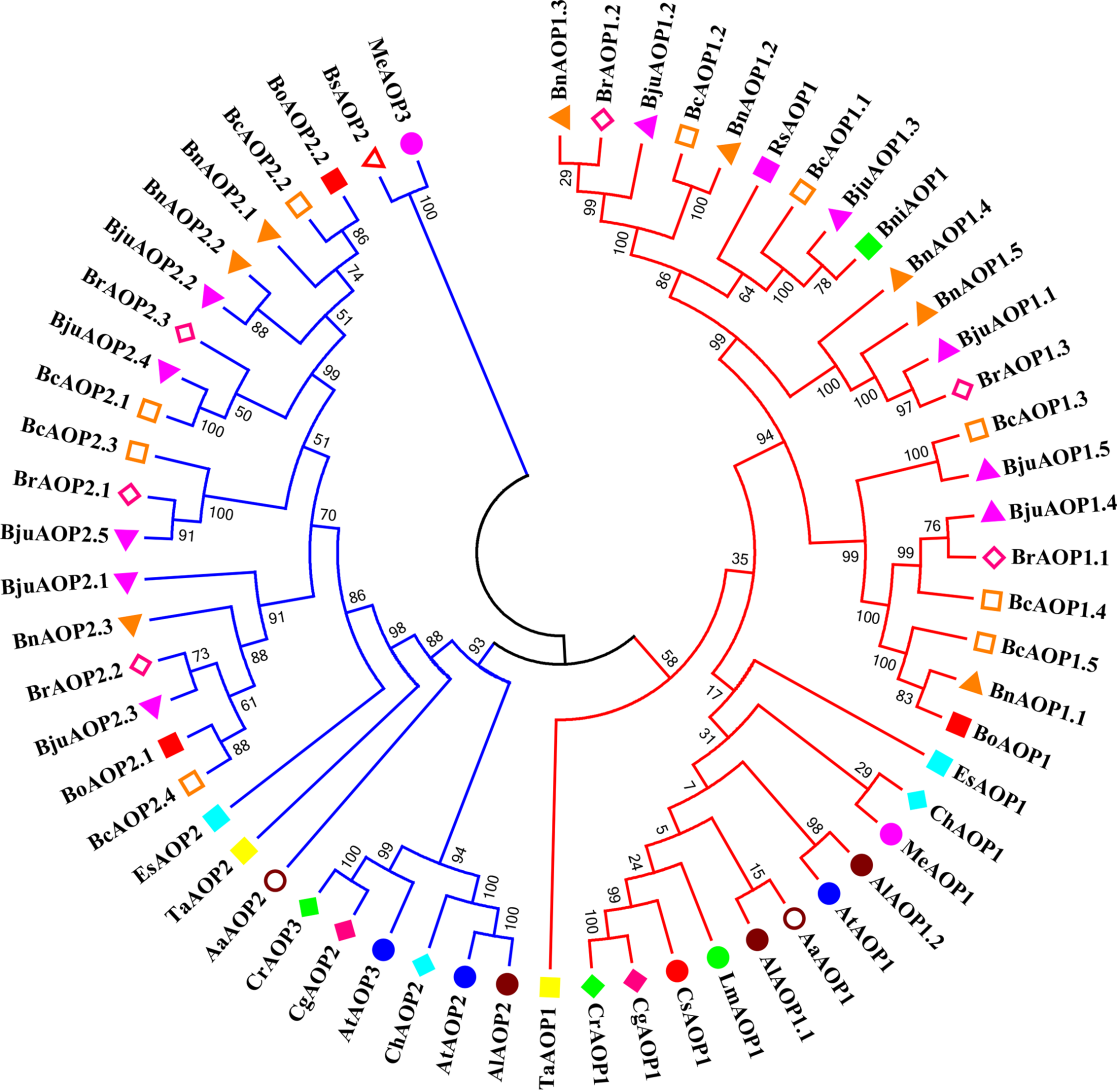
Phylogenetic tree of AOP genes from Brassicaceae species. The phylogenetic tree was built using the neighbor-joining (NJ) method. The unrooted tree was generated using Clustal W in MEGA11 *via* AOP amino acid sequences from 19 sequenced Brassicaceae species, including: *Arabis alpina* (*Aa.*), *Arabidopsis lyrate* (*Al.*), *Arabidopsis thaliana* (*At.*), *Brassica carinata* (*Bc.*), *Brassica juncea* (*Bju.*), *Brassica napus* (*Bn.*), *Brassica nigra* (*Bni.*), *Brassica oleracea* (*Bo.*), *Brassica rapa* (*Br.*), *Boechera stricta* (*Bs.*), *Capsella grandiflora* (*Cg.*), *Cardamine hirsute* (*Ch.*), *Capsella rubella* (*Cr.*), *Eutrema salsugineum* (*Es.*), *Lepidium meyenii* (*Lm.*), *Microthlaspi erraticum* (*Me.*), *Raphanus sativus* (*Rs.*), and *Thlaspi arvense* (*Ta.*). Genes from each species were labeled with different colors. The numbers at the nodes represent bootstrap percentage values.

An *AOP* gene evolutionary tree was constructed for TSM and 18 other cruciferous crops that have been sequenced to further understand the evolutionary origins of the five members of the *BjuAOP2* gene family in TSM (**Figure 2**; **Supplementary Table S4**). The *AOP* gene family is divided into two major categories: (1) *AOP1s* consisting of 33 *AOP1* amino acid sequences; (2) *AOP2s* and *AOP3s* consisting of 25 *AOP2* amino acids and 3 *AOP3* amino acid sequences. In addition, small branches (N ≤ 3) formed by the genes are all derived from different Brassicaceae species, and the small branches further form large branches in the form of clusters containing two and three genes. Members of the TSM *AOP* gene family are more similar to those in *B. carinata* and *B. rapa* than those in *Arabidopsis thaliana*. The TSM *BjuAOP2* gene has the closest affinity to *B. rapa*, and *B. nigra*, which is consistent with the evolutionary origin doctrine in U’s Triangle Brassica Species (Cheng et al., 2016).

**Figure 3:**
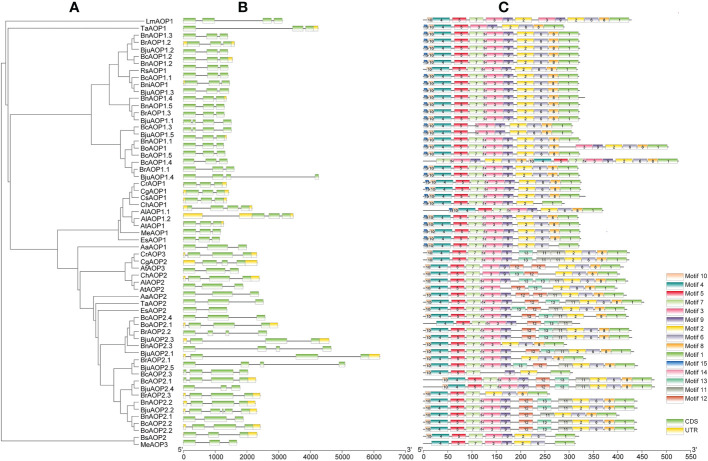
Gene structures and motifs of 61 *AOP* genes identified in *B juncea* and other sequenced Brassicaceae crops. **(A)** unrooted phylogenetic relationships among the AOP protein sequences. **(B)** exon-intron organization of corresponding AOP gene. Exons are represented by green boxes, while introns are represented by gray lines. **(C)** conserved motifs of AOP genes. Colored boxes indicated conserved motifs and gray lines represent non-conserved sequences. The length of motifs in each protein was showed proportionally.

**Figure 4:**
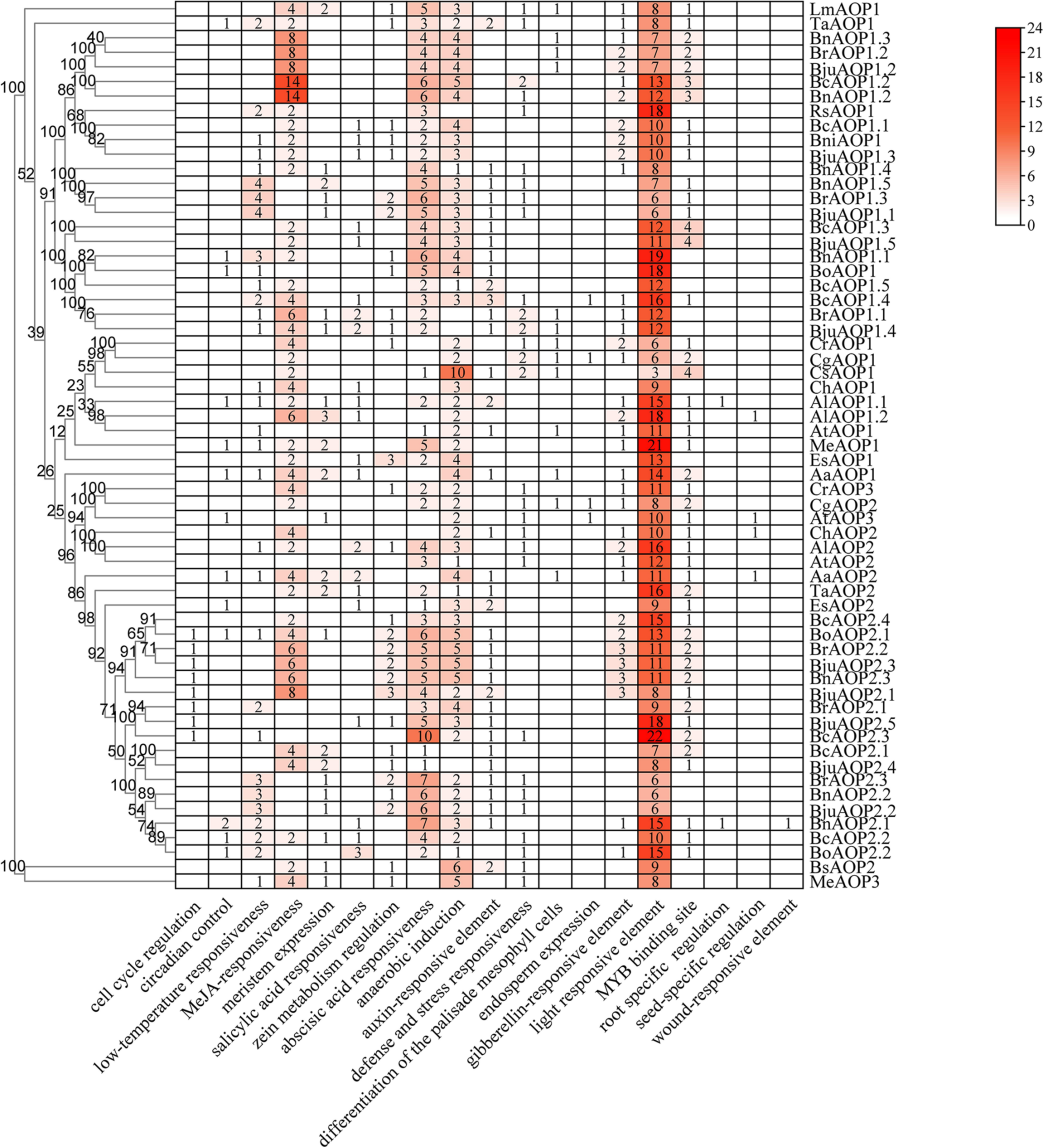
Cis-acting regulatory elements in the promoter region of *AOP* genes. The numbers and the depth of red represent the frequency of the elements that occur in the promoter region.

The gene structure diagram from the *AOP* gene family (**Figure 3A**) revealed that the *AOP* family is conserved in the number of exons, 53 of the 61 members containing three exons. Despite the similar exon length of most genes, the intron lengths differed significantly. The *AOP* family was analyzed for conserved motifs using the MEME online tool (**Figure 3B**). In total, 15 conserved motifs of 8-41 aa in length were contained in the *AOP* gene (**Figure 3C**; **Supplementary Table S5**). In addition, the structural domains of 15 motifs were analyzed using NCBI-CDD online software. The motif 10,4,5 and 7 together form the DIOX_N domain. And the motif 2, 6, 8 and 1 together form the 2OG-FeII_Oxy domain structural of the *AOP* gene family.To further elucidate the functional element that influences expression of *AOP* genes, the promoter sequences were analyzed using the PlantCARE database to identify cis-regulatory elements in the promoter region. Nineteen types of stress- and hormone-related cis-acting regulatory elements were detected in the promoters of *AOP* genes (**Figure 4**; **Supplementary Table S6**). All 61 *AOP* genes contained element related to light. The MYB bingding site were detected in 47. Hormone-related elements (MeJA-responsiveness, salicylic acid responsiveness, zein metabolism regulation, abscisic acid responsiveness, auxin-responsive element, gibberellin-responsive element) were detected in more than 45 members of *AOP* genes. Other results are shown in the **Figure 4**.

### 3.3 Subcellular localization of the protein encoded by the *BjuAOP2* gene

**Figure 5:**
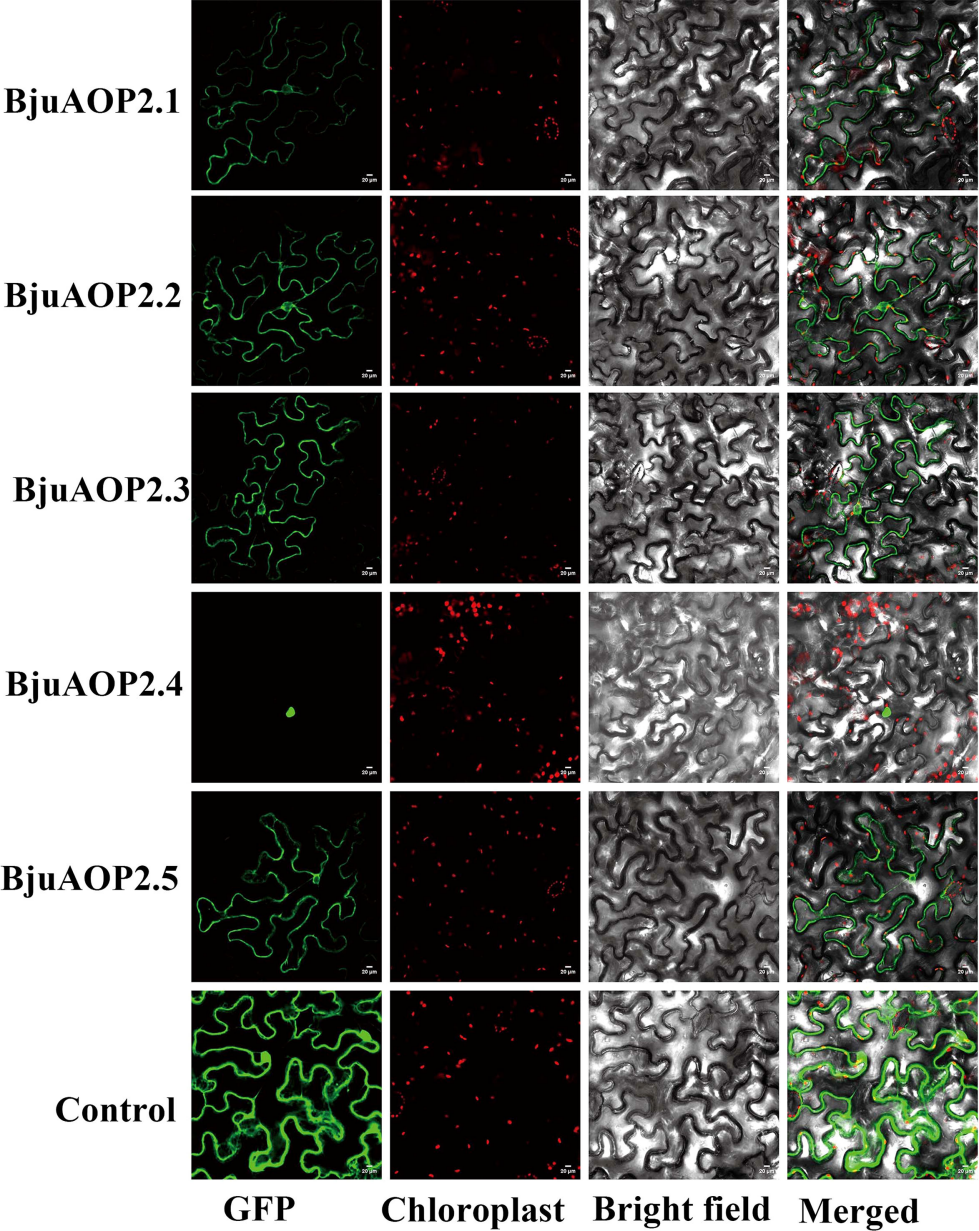
Subcellular localization of BjuAOP2 proteins in *Nicotiana benthamiana*. These genes were fused with the GFP protein driven by the 35S promoter. Green fluorescence and chloroplast autofluorescence (red) images were captured in a dark field; however, a bright field was used to get images of cell appearance. Bar=20 μm.

The green fluorescent protein (GFP) fusion transient expression vector containing the *BjuAOP2* gene was injected into tobacco leaves and observed by laser confocal microscopy (**Figure 5**). Except for *BjuAOP2.4*, where a green fluorescent signal was observed only in the nucleus, the other four family members were observed to fluoresce in both the cytoplasm and nucleus. Thus, *BjuAOP2.1*, *BjuAOP2.2*, *BjuAOP2.3*, and *BjuAOP2.5* were expressed in the cytoplasm and nucleus, and *BjuAOP2.4* was expressed only in the nucleus, consistent with the prediction results of subcellular localization.

### 3.4 Expression pattern of the *BjuAOP2* gene

**Figure 6:**
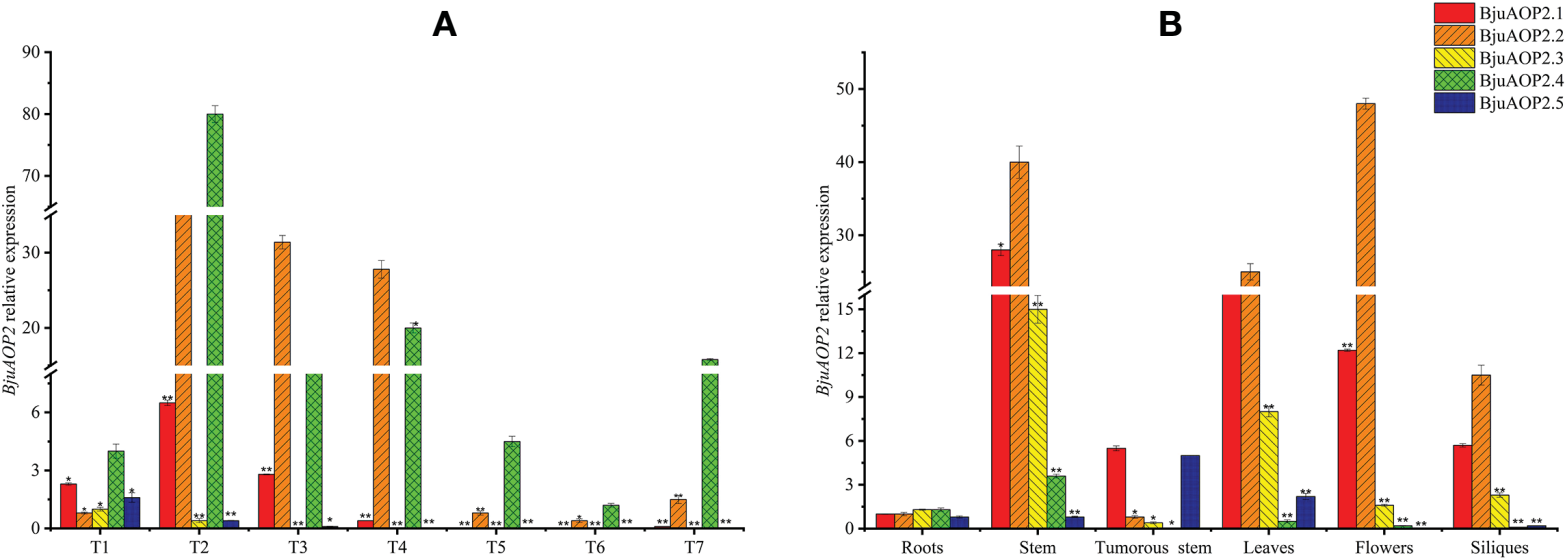
Expression pattern of *BjuAOP2* in different tissues and different developmental periods. The vertical axis indicates gene expression levels relative to *BjuAOP2.5* expression in roots **(A)** and *BjuAOP2.2* expression in 4 weeks after sowing. T1-T7 represent the seven times 4, 7, 10, 13, 16, 19, and 22 weeks after sowing **(B)**. Error bars represent the standard deviation from three biological repeats. * and ** indicate significant differences at P<0.05 and P<0.01 using ANOVA analysis followed by a Duncan test, respectively.

The expression patterns of five *BjAOP2* genes in various tissue parts of B186 and various developmental stages of the tumorous stem were analyzed using real-time PCR (**Figure 6**). Expression of *BjuAOP2* genes was detected in roots, stems, tumorous stems, leaves, flowers and siliques. *BjuAOP2* genes had significantly different expression patterns in different tissues (**Figure 6A**). *BjuAOP2.2* were more highly expressed in flowers than in other tissues examined (**Figure 6A**), whereas *BjuAOP2.1*, *BjuAOP2.3* and *BjuAOP2.4* were more highly expressed in stems than in other tissues (**Figure 6A**). *BjuAOP2.5* were more highly expressed in tumorous stems than in other tissues (**Figure 6A**). Analysis of the expression pattern of the *BjuAOP2* gene at different stages of tumorous stem development revealed different expression patterns with the expansion of TSM (**Figure 6B**). The expression of *BjuAOP2.1*, *BjuAOP2.2* and *BjuAOP2.4* peaked at 7 weeks after sowing before falling back to basal levels. *BjuAOP2.3* and *BjuAOP2.5* showed low expression level throughout the whole growth period.

### 3.5 *In vitro* enzyme activity analysis of *BjuAOP2*

**Figure 7:**
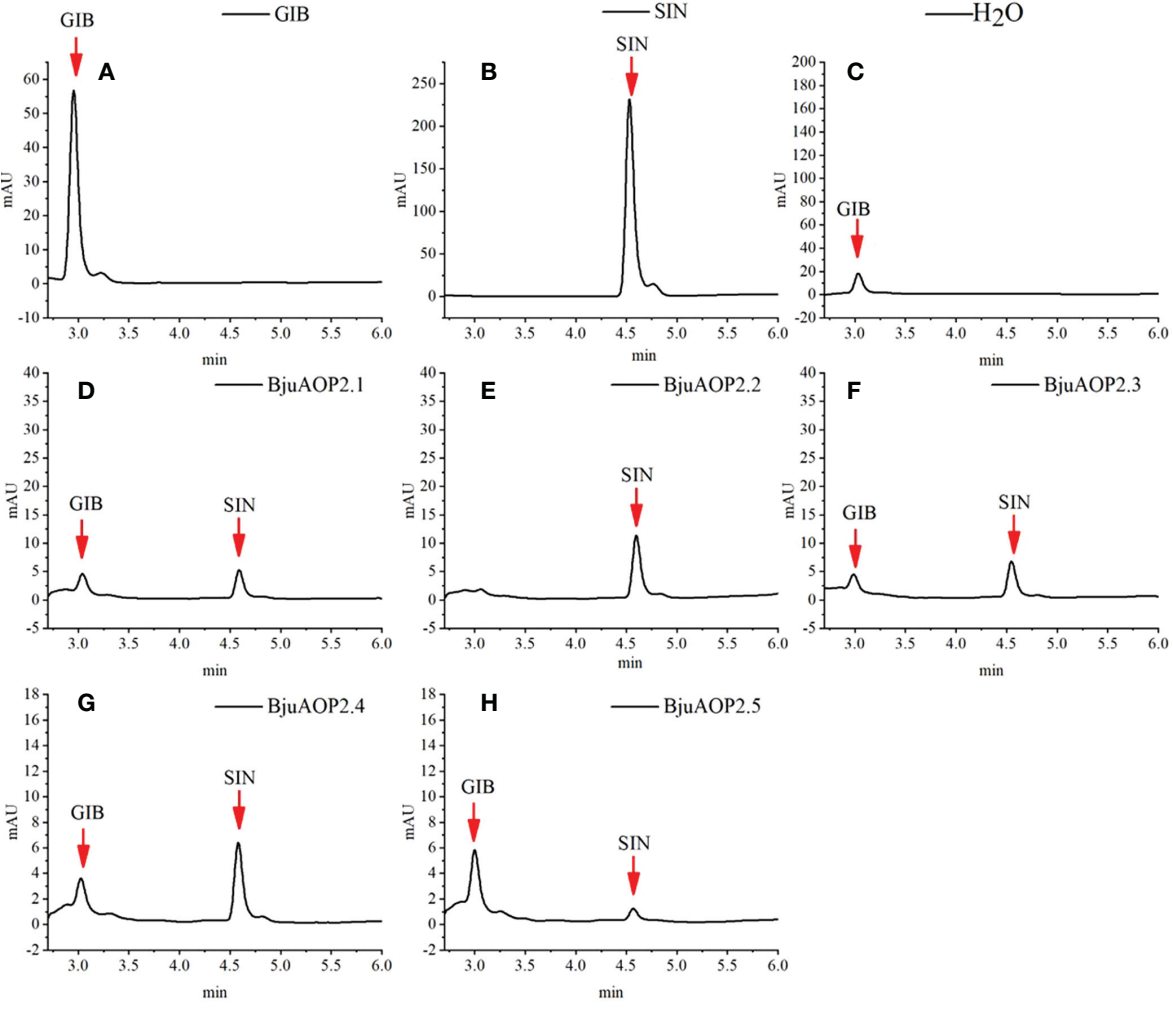
Enzymatic activity of heterologously expressed *BjuAOP2*. The purified desulfoglucosinolates extracted from *E. coli* were subjected to HPLC (monitored at 229 nm) and results were documented. **(A)** indicates desulfated GIB (glucoiberin) standard, **(B)** indicates desulfated SIN (sinigrin) standard, **(C)** indicates desulfated GIB standard treated with ddH_2_O as the negative control; **(D–H)** indicate desulfated GIB standard treated with *BjuAOP2.1, BjuAOP2.2, BjuAOP2.3, BjuAOP2.4* and *BjuAOP2.5* enzymes, respectively.

*BjuAOP2* prokaryotic expression vector was constructed using PET-32a. The protein expression was induced by IPTG, and the *BjuAOP2* fusion protein was obtained by the His-tag purification column. *In vitro* enzymatic activity analysis of five BjuAOP2 fusion proteins was performed using GIB as substrate. As shown in **Figure 7**, all five BjuAOP2 proteins successfully catalysed the conversion of GIB to SIN. The standards GIB and SIN were detected at 3.3 min and 4.6 min, where only GIB was detected in the control group. However, both GIB and SIN were detected in the protein elutes of all five members. Therefore, all five *BjuAOP2* proteins of TSM have catalytic activity.

## 4 Discussion

Increasing the beneficial GSL content has been a major breeding goal for Brassica species in recent years (Ishida et al., 2014; Barba et al., 2016). The hydrolysis products of GRA such as isothiocyanates have anticancer effects in humans (Fahey et al., 2001; Fahey et al., 2002). Furthermore, enzymatic degradation of GRA to PRO inhibits iodine uptake in mammals, leading to thyroid dysfunction. The high content of SIN in TSM adds a strong mustard spiciness, which is derived from the degradation of GIB by the *AOP2* gene. Therefore, the focus of breeders’ attention has been the buildup of beneficial GSL components and contents in Brassica vegetables and the reduction of harmful GSL components and contents. In *B. juncea*, Augustine and Bisht (2015) used RNA interference technology to accumulate a large amount of GRA, a beneficial GSL product, by affecting the expression of *AOP2*, indicating the significance of *AOP* genes in the breeding improvement of Brassica. The recent publication of the *B. carinata* reference genome announced the completion of the reference genome assembly for the U’s Triangle Brassica Species (Song et al., 2021), which facilitates the study of the functional and evolutionary relationships of *AOP* genes. In this study, five *BjuAOP2* genes in TSM were identified and cloned. All five genes possess biological catalytic activity, but the expression and subcellular localization results are tissue-specific and differential, respectively. Therefore, functional divergence may exist in the regulation of SIN synthesis by these five genes. Overall, the findings of this study may provide a reference for breeders to improve the TSM GSL fraction and content.

As a family with large number of members, the Cruciferae contains 338 genera and 3709 species covering many crops that have economic importance (Warwick et al., 2006). The core crops of the Cruciferae family underwent genomic triploidization events during evolution (Lysak et al., 2005). Theoretically, the number of TSM *BjuAOP2* should be three times higher (six) than that of *Arabidopsis thaliana*. However, only five *BjuAOP2* family members were retained during the evolution of TSM, indicating the loss of *BjuAOP2* genes in this process. The evolutionary analysis of cruciferous *AOP* genes in this study revealed that *AOP1* was in a separate group (**Figure 2**). Previous studies have shown that the *AOP* locus in cruciferous species underwent two gene duplication events. The first caused the divergence between *AOP1* and *AOP2/3* (the *AOP1* gene was the ancestor gene of the *AOP2/3* gene) while the second event led to the formation of the AOP2 and AOP3 genes (Kliebenstein et al., 2001a). Subsequently, some *AOP* genes were lost due to the formation of new species or adaptation to variable environments.

Gene duplication enables the tissue-specific expression of genes that undergo replication in response to variable environmental stimuli; in addition, the replicated genes have more diverse expression patterns relative to single genes (Li et al., 2005; Kliebenstein, 2008; Huang et al., 2022). The five *BjuAOP2* genes in this study exhibited expression divergence in different tissues and different periods of tumorous stem development, indicating varied regulation of the genes. The *BjuAOP2* gene is expressed in trace amounts in roots and in higher amounts in leaves and stems in B186 (**Figure 6A**), depicting congruency with the expression pattern of the *AOP2* gene in *Arabidopsis thaliana* (Neal et al., 2010). Subcellular localization analysis indicated differences in the expression sites of these five genes. The promoter regions of the *BjuAOP2* genes were further analyzed using PLACE software, and each of them had some specific cis-acting elements (**Figure 4**). These elements were associated with tissue-dependent expression, hormone, biotic and abiotic stress responses, resulting in differential expression of the five *BjuAOP2* genes in various tissues and developmental periods. Thereby leading to differences in GSL accumulation in TSM thus improving plant adaptation to the external environment.

Genes may undergo depletion, defunctionalization, functional maintenance, or functional differentiation across copies during replication (Zhang, 2003). In this research, *in vitro* experiments of prokaryotic expression demonstrated that all five genes in TSM can convert GIB to SIN, leading to abundant SIN in TSM. Therefore, all five genes have biocatalytic activity (**Figure 7**). The multiple-sequence alignment of the five *BjuAOP2* proteins showed that they have two typically conserved structural domains at both their C- and N-terminal ends, whereas the sequence variation outside the conserved structural domain is large (**Figure 1**). Studies in *B. oleracea*, *B. rapa*, and *Arabidopsis thaliana* have revealed that disruption of the 2OG-FeII_Oxy structural domain leads to loss of *AOP2* gene function (Li and Quiros, 2002; Neal et al., 2010; Zhang et al., 2015). The integrity of the 2OG-FeII_Oxy structural domain may be critical for the *AOP2* protein to maintain its catalytic activity in a variable environment.

This research dealt with the elucidation of the expression characteristics of the *BjuAOP2* gene in TSM and its role in the GSL biosynthesis pathway. In addition, the evolutionary relationships of this gene were analyzed. These findings provide a guide for breeders to improve the aliphatic GSL component of TSM by traditional breeding methods or genetic modification methods.

## 5 Conclusion

This work identified five *BjuAOP2* genes from the TSM genome that were cloned, bioinformatically analyzed, and validated for *in vitro* activity. First, the protein sequences of the genes were analyzed. All five genes in TSM possessed two conserved structural domains, with increased variation in the variable regions, indicating functional divergence. Subsequently, the evolution of *AOP* genes in cruciferous species was analyzed. All *AOP1* genes clustered into a large independent branch, whereas *AOP2* and *AOP3* were clustered into a separate large branch. The *in vitro* activity analysis verified the biocatalytic activity of these five *BjuAOP2* genes. In addition, their expression pattern analysis suggested variation in different growth periods or different tissues. Finally, tobacco injection-based subcellular localization analysis using transient expression assay indicated the differences in the cellular localization of these five *BjuAOP2* genes.

## Data availability statement

The datasets presented in this study can be found in online repositories. The names of the repository/repositories and accession number(s) can be found in the article/**Supplementary Material**.

## Author contributions

Conceived and designed the experiments: JC, BC and YiL. Performed the experiments: BC, YuL, DZ, CX and ZL. Analyzed the data: JC, BC and YiL. Funding acquisition, Project administration: JC and YiL. Writing, reviewing and editing: JC, BC and YiL. All authors contributed to the article and approved the submitted version.
